# Understanding factors for adhering to health recommendations during COVID-19 among older adults - a qualitative interview study using health belief model as analytical framework

**DOI:** 10.1186/s12877-024-05132-9

**Published:** 2024-06-19

**Authors:** Johanna Gustavsson, Linda Beckman

**Affiliations:** 1 Department of Political, Historical, Religious and Cultural Studies, Karlstad University, Universitetsgatan 2, Karlstad, 651 88 Sweden; 2 Centre for Societal Risk Research, Karlstad University, Universitetsgatan 2, Karlstad, 651 88 Sweden; 3 Health Science, Karlstad University, Universitetsgatan 2, Karlstad, 651 88 Sweden; 4 Health Service Research, Management & Policy, University of Florida, 1125 Center Dr, Gainesville, FL US

**Keywords:** Mental health, Health Belief Model, Geriatrics, Qualitative, Quality of life, Sweden

## Abstract

**Introduction:**

The COVID-19 pandemic has presented a unique possibility to broaden the understanding of people’s reactions to a global crisis. Early on, it became evident that older adults were particularly vulnerable to the virus and that the actions of this age group would be crucial to the outcome. This qualitative interview study uses the Health Belief Model (HBM) framework as an analytical tool to examine older people’s experiences of adherence to recommendations during the initial phase of the COVID-19 pandemic. It is important to view this study in the context of Sweden’s voluntary restrictions, which further highlight the unique nature of this research.

**Method:**

In April-May 2020, 41 adults aged 70–85 participated in unstructured phone interviews. The objective was to investigate older adults’ perceptions of the COVID-19 pandemic, particularly their understanding of the disease and the conditions that influenced their adherence to health recommendations. HBM was used as an analytical framework to guide the analysis of the interviews.

**Results:**

Despite perceiving COVID-19 as a severe threat to health and society, participants did not let fear dominate their responses. Instead, they demonstrated remarkable resilience and a proactive approach. For some, the perceived susceptibility to the disease was the primary motivator for adherence to the Swedish national recommendations. Notably, trust in the authorities and family members’ requests significantly bolstered adherence. Moreover, adherence was found to contribute to feelings of safety. Conversely, potential barriers to adhering to recommendations included missing loved ones and frustration with sometimes ambiguous information from authorities.

**Conclusions:**

The results from this study indicate that older adults are willing to adhere to voluntary restrictions during a global pandemic. Relatives of older people are a resource for communicating information regarding safety and health messages, a message that is preferably thorough and consistent. Further, much can be gained if loneliness during isolation can be mitigated since missing loved ones appears to be a potential barrier to adherence.

**Supplementary Information:**

The online version contains supplementary material available at 10.1186/s12877-024-05132-9.

## Background

Right from the start of the COVID-19 pandemic in March 2020 (WHO, 2020), it was evident that people over 70 were severely affected in terms of the severity of symptoms, need for intensive care, and death [1]. In the pandemic’s initial phase, non-pharmaceutical interventions (NPI) were the only strategy to limit spread. In Sweden, the authorities chose a unique strategy for protecting risk groups and limiting transmission, including only recommended restrictions and not practicing lockdowns. The official Swedish strategy aimed to reduce the spread of the coronavirus to limit the strain on the healthcare system and protect vulnerable groups such as older people and other risk groups [2]. Compared to the other Nordic countries (Norway, Finland, and Denmark) that practiced an elimination strategy, Swedish restrictions (mitigation strategy) were initially much less strict than those in other Nordic countries. For example, measures including lowering the number of participants allowed in public gatherings, restrictions of entry into the country, and restrictions regarding visits to care homes for older people were fewer and weaker in Sweden than in the other Nordic countries [3]. During the first year of the pandemic, COVID-19 mortality per capita was ten times higher in Sweden than in Finland and Norway and five times higher in Denmark during the same period [4]. When the Swedish strategy was scrutinized, it was, in conclusion, clear that the care system for older people during the pandemic had failed. The main reasons were a high general spread of the infection, lack of protective equipment in residential care, and problems with organization and staffing. The investigation concluded that too few, too late, and too weak measures were implemented, resulting in the enhanced spread of the coronavirus [4–6].

During the first month of the pandemic, people over the age of 70 were recommended to take more extraordinary precautions than the rest of the population and practice strict social distancing, avoiding all interactions outside of the household [7]. The incentives for adherence to recommendations communicated by the government authorities were primarily twofold: that the novel coronavirus is a threat to one’s health and that the current situation caused substantial stress to the healthcare system. In addition, the collective responsibility to mitigate the consequences was emphasized. The rationale is that the risks could be monitored by following the recommendations, and the societal consequences are limited. Based on the different strategies (mitigation or elimination strategy), a hypothesis is that countries adopting the loose mitigation strategy would spare peoples’ mental health. Aknin and Andretti [8] investigated the differences between these two strategies, revealing small changes in mental health during the first 15 months of the COVID-19 pandemic and that more stringent COVID-19 policies were associated with poorer mental health. This was also true for the comparison of Sweden against the other Nordic countries.

In old age, a person must deal with the physical, social, and cognitive limitations of aging. This, and experiences during a long life, can affect the perception of crises and the associated health recommendations. During previous pandemic outbreaks, evidence indicates higher adherence to NPI in old age [9]. However, for the COVID-19 pandemic, unique in scale and duration, a review looking at NPI found that older adults were less likely to adhere [10], and another recent study shows that the differences in perceived risk of health consequences of COVID-19 between middle-aged and older adults are minor [11].

Previous research from Western countries using qualitative research to investigate adherence to NPI during COVID-19, primarily focusing on older people, is relatively scarce. Studies including a mix of younger and older people found that reasons for non-adherence to COVID-19 guidelines included lack of trust in the government and confusion or lack of knowledge about rules, with a reduced risk perception relating to the upcoming vaccine or being at a younger age [12–14], “alert fatigue,” i.e., feelings of impossibility to keep up with the rules, feeling “sick of it.”, helplessness, resistance (i.e., people are fed up and will break the rules) [14], lacking control of practicing distance, wanting to do voluntary work, support from friends to stay at home or jointly bending the rules, and perceived lack of adherence in the local area compared to the neighborhood [12–14].

There is a growing body of quantitative research on adherence to health-promoting recommendations regarding COVID-19 [15]. Survey data identified aspects that affect adherence to NPI, e.g., knowledge and perceived severity and control [16]. However, results that are restricted to data from the questions asked in the surveys might miss explaining why these factors are essential. Hence, the potential for understanding the underlying factors affecting barriers and facilitating factors is limited compared to qualitative studies. This study used qualitative interview data to deepen the understanding of older adults’ health behavior during the COVID-19 pandemic.

Another identified gap in the present literature on the subject is theory-driven studies [17]. Adherence to NPIs often requires people to change everyday behavior. Frameworks and behavior change theories can be used to categorize factors that might affect adherence. The Health Belief Model (HBM), among several other models, is developed to understand why people adapt or fail to adapt to health-promoting behaviors. The HBM suggests that people’s understanding of the health threat, perceived benefits of action, potentially harmful aspects of action, and belief in the ability to action can explain engagement (or lack of engagement) in health-promoting behavior [18–21]. The theoretical constructs of the HBM include perceived susceptibility and severity of the threat, as well as perceived benefits and perceived barriers to action. Further, external factors that can influence behavior, i.e., cues to actions, individual characteristics, and, finally, self-efficacy, are included in the model.

In summary, the COVID-19 pandemic offers an extreme and novel situation, and it is essential to investigate people’s responses and reactions to the situation to improve preparedness and the ability to handle similar crises in the future. Previous research on the subject is mainly based on survey data; thus, there is a need for qualitative data that can deepen the understanding of barriers and facilitators for adherence. Also, surprisingly, little research has been done on older people in this matter. Therefore, this qualitative interview study aims to explore and investigate older people’s experiences of adherence to recommendations during the initial phase of the COVID-19 pandemic, using the HBM framework as an analytical tool. As far as we know, this is the first study to study adherence to NFI during the COVID-19 pandemic in a Swedish context among the primary risk group of older adults.

## Materials and methods

In this qualitative study, we conducted interviews in two phases. The first set of interviews (*n* = 41) was conducted in April-May 2020. A sub-sample of individuals was randomly drawn from the first phase group (*n* = 9) in the second phase, conducted in November-December 2020. This interview study is part of a project where we, apart from the interviews, also collected data via a questionnaire (*n* = 1854). The questionnaire included questions on mental health status, risk perception, and compliance with recommendations, and the interviews were set to dig deeper into these aspects. Results from the questionnaire can be seen in Authors (*blinded xxxx*). The study is reported according to the COnsolidated criteria for REporting Qualitative Research (COREQ) checklist [22]. The study was approved by the Swedish Ethical Review Authority (No. 2020 − 01600). Informed consent to participate in the study was obtained from all subjects.

### Participants and recruitment

Interviewees were recruited via ads on Facebook (25% of participants) and through a national pensioners’ organization. For the Facebook recruitment, a Facebook page was set up with links where eligible participants could contact us to signal their interest and leave contact information. We also used snowball sampling, asking participants if they knew anyone willing to participate in the study and if they could ask that person to contact us. The other recruitment source was a national pensioners organization that informed their members about possibly participating in a study in one of their semi-weekly information letters. A link to a parallel survey (authors blinded 20xx) was provided in the letter. In that survey, we asked for consent to participate in an additional interview, and over a hundred people volunteered. After conducting several interviews, we chose to seek out a subsample of those who reported feeling anxious about the current situation in the survey (*n* = 6). This was to obtain more variety in our sample, as the initial interviewees had been handling the situation calmly and patiently, and we wanted to know if there were people with other views. In addition, it was a way to avoid missing a more anxious group and potentially less willing to participate in a study. The inclusion criteria were age 70 or older, and the exclusion criteria were non-Swedish speakers.

The participants were between 70 and 84 years old, with a majority between 70 and 75, consisting of 33% men. In total, 27% of the participants lived alone, 20% lived in housing without a garden, i.e., apartment, and rated their health between 6 and 10 (m 8.5) on a scale of 1–10. One-third lived in a major city or suburb, one-third in a smaller city, and one-third in the countryside. Examples of professions were teacher, nurse, academic, farmer, entrepreneur, banker, and social worker. One-third were partly still working.

### Interviews

For the individual in-depth telephone interviews, which lasted around 30–45 min each, we used a semi-structured interview guide and, with an inductive approach, asked about their daily life and experiences of the present situation. The interview guide had four themes: perception of recommendations, information, risk, and health, all regarding the COVID-19 pandemic (interview guide appendix 1). In the follow-up interviews, we again asked them to describe their daily life, view of the situation, and changes during the over half a year of living with restrictions.

### Analysis

The authors performed the analyses jointly. The interviews were transcribed verbatim and read through several times by both authors. An abductive approach was taken, and we structured the categories using the theoretical constructs from the HBM. Using the method for content analyses [23], the data was first coded by identifying meaning units, advancing to identifying patterns according to the constructs of the HBM (for an example of the analytical process, see Appendix 2). During the interviews, we asked how the interviewees perceived the threat.

Regarding perceived benefits or barriers, the interviewees were not asked directly. Instead, the latent content expressing these aspects was identified in the analytical process. Illuminating quotes were used to exemplify closeness to data, and the quotes are coded so that for the last of the three digits, one equals the first round of interviews and two the second.

## Results

The results are structured by theoretical constructs of the HBM: *Perceived susceptibility to and severity of the health threat, Perceived likelihood of reducing the threat by engaging in the behavior, Potential barriers or costs to adhering to recommendations*, and *Cues to action.* The construct of *self-efficacy* was interpreted throughout the results as trust in the ability to cope with the situation, and this concept was further explored in the discussion section.

### Perceived susceptibility to and severity of the health threat

It emerged from the interviewees that the virus and the illness it caused were perceived as a severe threat to health and society. There was a fear of containing the virus and thus getting ill, indicating some level of susceptibility. However, for most, it seemed like the fear was not taking over or ruling their thoughts. Instead, they dealt with the risk of getting a potentially deadly disease by acknowledging the risk but not letting fear take over; as one person said:

*It’s in the back of my mind; this is how it is.…we have to be able to live a reasonably normal life together, my husband and I. I’m not going to dwell on this (1.15.1).*

This view was even more prevalent in the second round of interviews 7–8 months later.

For some, it was apparent that perceived susceptibility to the disease was the main reason to adhere to the national recommendations. They expressed not being worried, but at the same time, they were taking substantial precautions. One woman expressed her dual feelings and measures:

*It’s not like panic. I do not feel scared. I spray some [with alco gel] on door handles and the drum floor. So, I’m careful with that. I wash my hands a lot. But I do not panic. Maybe I should be more scared than I am. (1.8.1)*

However, despite susceptibility to the virus, some raised the concern that compared to other health risks, such as illness and injuries, COVID-19 was judged less likely to be a threat and, simultaneously, beyond their control.

*Sometimes, I think it’s better to get it over with. You don’t want to become seriously ill, but you cannot choose; you don’t know how sick you will get (1.11.1).*

*Well, if I get sick, I get sick; if I get a stroke, I get a stroke (2.19.1)*.

This view could possibly contribute to a less strict adherence to the recommendations, as not seeing oneself within the risk group can be an obstacle.

The follow-up interviews show that accepting the situation was more widespread. In the first round of interviews, one participant said that it is easy to fall back into old habits and forget the current recommendations:

*I’ve been shopping for groceries but have kept my distance when walking. You should keep your distance, but you fall back into old habits before you know it. (1.7.1).*

In the follow-up interview, the same person acknowledged that following recommendations has now become the new norm:

*You have gotten used to the pandemic in some ways. You accept the situation; it is what it is (1.7.2).*

Several of our quotations follow this reasoning, showing that the older people in our study managed the situation quite well.

In summary, despite the participants’ feelings of general good health, their subjective perception of the risk of acquiring COVID-19 seemed rationalized high, which, according to the HBM, would suggest high adherence to the recommendations.

### Perceived benefits and the likelihood of reducing the threat by engaging in the behavior

The interviewees expressed confidence in the efficiency of the recommendations, and it was evident that engaging in a behavior in line with the recommendations provided a feeling of safety. The message from government authorities was perceived as clear, sufficient, and reliable, and its representatives were attributed with an elevated position. For some, the Swedish Public Health Agency was exceptionally trustworthy.

*I followed everything closely, and yes, I think the state epidemiologist is right. I’m pretty sure of that. (2.2.1).*

When adhering to recommendations, they felt protected. If they are less rigid with adherence, they find ways to protect themselves and contain a feeling of security, keeping the risk in mind and still doing everyday things in an adjusted manner.

*When we go shopping, we are careful with distance, no problems as there are few customers, and we only do the most necessary, very careful not to touch our face when we are in town and wash our hands very carefully when we get home. You always have this in the back of your mind, that social interaction simply has to take place from a distance (1.14.1).*

Another example is when the need to hug a grandchild becomes overwhelming; a woman solves the matter by hugging from behind, avoiding contact, or breathing.

Adhering was also seen as a way of mitigating the situations on a societal level.

*The government says that everyone will get COVID-19 but that you have to postpone your illness so that healthcare can manage it; that’s how I feel (2.21.1).*

The understanding was that following the recommendations would ensure everything would be fine and the situation would not get out of control.

It emerged that many interviewees felt healthy and had trouble viewing themselves as old and vulnerable. Still, there was knowledge and realization that they could get seriously ill, which was frightening and made them practice social distancing. One person said:

*I feel really healthy. But I would not want to catch it [COVID-19] anyway. I think that would be awful, so we both stay home (2.16.1).*

Hence, the participants seemed confident that engaging in the behavior would reduce the threat.

### Potential barriers or costs to adhering to recommendations

A barrier in adhering to recommendations was the heartache of longing for loved ones, especially grandchildren. Being a part of the children’s lives was necessary, and missing months, or even years, could seem unbearable.

*I am very active with one of my grandchildren; it is a sadness [not to be able to meet]. It’s not like I sit at home and bury myself in misery; it’s a sadness in my heart. I try to be active anyway and have a lot of phone contact, but it’s not the same (2.12.1).*

This is also related to the longing for physical contact, missing hugs, and sitting close to loved ones. For the first time, some had realized what role social interaction plays in their lives and how much they needed and missed it.

Another potential barrier was the boredom that social isolation brings. The recommendations were easy to understand and follow, but every day tended to be the same, and life became boring. Some felt apathy, and even if they had all the time in the world, it could be hard to get started with something meaningful. One person said:

*You live in a vacuum, somehow unable to make plans. (1.7.1).*

The dullness was tearing, and they expressed a risk that they would get too sick and tired of social distancing and eventually give up.

As mentioned earlier, the interviewees perceived the government authorities’ message as clear, sufficient, and reliable. However, even though stating that information was sufficient, it was evident that there were question marks regarding the recommendations. The initial message about herd immunity and that the spread of the virus was under control was puzzling and contradictory. Doubts about the vast differences in how the Swedish authorities handled the situation compared to most other countries, e.g., regarding face masks, immunity, and information, were also expressed. In the follow-up interviews, some had lost their trust in the authorities:

*I’m just saying I have completely lost confidence in the FHM [Public Health Agency of Sweden]. (2.7.2).*

Some even had disbelief that the recommendations were there to protect them. Nevertheless, they emphasized that they were still determined to adhere to the recommendations. A strong argument was that uncertainty regarding what is correct only leaves one option: to depend on the authorities.

A recurring opinion was resisting older adults being addressed as one homogenous group. Some accept being described as a vulnerable group and thought it was logical as the virus hit them harder. Others were upset and rejected being described as fragile and associated with old age and felt that it did not concern them:

*It was like -am I suddenly old now? Of course, I am, but, it was a bit strange. I think I am pretty healthy and go out and about as usual. But you are not supposed to. I don’t understand. (1.3.1).*

This feeling of not belonging to a risk group could be a barrier to adherence.

In summary, boredom, contradictious messages, and being addressed as one homogenous group could hinder adherence, but longing for relatives and friends was the most powerful. For very old or sick people, two years of social distancing could mean never being able to experience closeness before dying.

### Cues to action

Perhaps the most important cue to behavior change, or trigger factor, came from family members. The interviewees’ adult children and other close relations played a vital role in the adaptation process:

*The initiative to stop working, I have to admit, was not mine. My children told me that -enough is enough; you must give up working. (2.12.1).*

Friends and family set the terms for social interaction through signaling distance, e.g., holding up an item and visualizing the recommended physical distance. This was generally accepted. However, it also led to secrecy when the older adult chose to break the rules set up by others. Some even expressed anger (certainly mixed with fear) from their children:

*Yes, our eldest son called and scolded us and told us not to go shopping. (1.14.1).*

Another driving force was the potential for social shame if failing to comply with the recommendations. When pointed out as a group that should stay at home, wordings like “cheating” were used when stepping aside from the recommendations. As one participant puts it:

*Yes, it feels like, “Now I go here and do things no one knows.” It’s not often, but the times I’ve done it, it feels a little mischievous. It’s like sneaking around; you feel like a culprit. (2.9.1).*

This illustrates that the views of others, even if they are not close friends or relatives, influence behavior and have the potential to drive adherence.

## Discussion

As far as we know, this is the first study to investigate older peoples’ experiences of adhering to NPI in Sweden, which holds a unique strategy for protecting risk groups and limiting transmission. We used the HBM as an analytical tool to analyze the conditions for adherence to health-promoting recommendations among older adults initially and sometime into the COVID-19 pandemic. The results show that they accepted the situation and quite willingly adhered to limiting the pandemic’s effects on themselves and society. Our results show several aspects of how they perceive their situation and how that can affect behavioral adaptation, from which we can learn when managing future crises.

The response to risk has broadly been described in two ways: intuitive reactions to danger and logical reasoning [24]. Our interviewees’ reaction to the COVID-19 pandemic threat was primarily logical reasoning, although components of more emotional response were also seen, e.g., extensively worrying about others or excessive cleaning to avoid contamination. Further, when they compared the current risk to other health-related risks in old age, the perception of the severity of the virus decreased. This aligns with recent survey studies, indicating older adults’ emotional resilience to long-term crises [25, 26]. Perception of the threat generally has the weakest correlation to behavior change [27]. Based on survey data, Clark and Davila [28] found neither perceived susceptibility nor age-predicted adherence to health-protective behavior during the pandemic. Nevertheless, the results from this study indicate that the threat was taken seriously and, to some extent, did drive adherence even though it, especially over time, was seen as a manageable threat.

A further threat to adherence, also seen during the SARS outbreak 2002 [29], was the risk of stigmatization. Previous research argues that anticipated stigma might prohibit personal preventive behaviors during infectious diseases to avoid future stigmatization [29–31]. Even if evidence points to perceived benefits and barriers being the strongest predictor of behavior change [19, 32], cues to actions are essential, as seen in our results. In this case, adherence would probably be lower without external pressure from friends and family. Likewise, when addressing people over 70 collectively, older individuals who perceive themselves as healthy and “young” do not feel that the recommendations apply, and there is a risk of backlash. The heterogeneity of older adults and a lack of acknowledgment of this is a well-known factor for ageism [33]. In the context of COVID-19, addressing older adults as one frail unified group risks adding to stigmatization, reducing the older adults’ agency [34], and risking undermining the perceived liability in recommendations.

Perhaps the most severe barrier to adherence is the hardship of missing loved ones during social distancing, also identified as a central theme in other studies, including those of all ages [35, 36]. Here, an external cue comes into play - the view of friends and family, endorsing the older adults to adhere to the recommendation and practice social distancing. Previous literature suggests that family members are important for lifestyle behavior change in supporting and acting like role models [37, 38]. However, in the literature, we cannot find descriptions similar to our results of how older people’s grown-up children told their parents, with a strong emphasis on adhering to the recommendations. This shows how strong an impact children’s care can have. Concerning cues to action [19], it has been suggested that corporate knowledge of outbreaks, social acceptance, and perceived pressure from different sectors, including employers, mass media, government, and family, can all play a role in influencing adherence [15]. What makes the older adult hold on to the decision to adhere appears to be a combination of cues. Essential aspects are that recommendations were relatively easy to follow, sacrifices were perceived as mild, and the lifestyle when adhering became, or already was a habit. However, as seen in the results, a lack of clarity regarding information on government recommendations and the rationale for different rules could threaten trust in the government and potentially become a barrier to adherence. Studies on determinants of adherence to public health recommendations, based on survey and interview data, show that trust plays an important role. That mistrust can result in non-compliance [39], leading to seeking alternative information and hesitation to vaccinate. Different aspects of information and manifestation of infodemics, the term used for the extensive spread of disinformation and misinformation during a pandemic outbreak [40], are substantially researched. Although information flow is a cornerstone in managing pandemics, there seems to be a lack of data regarding older adults’ information behaviors and needs [41]. Older adults consumed a large amount of information from various sources during the COVID-19 pandemic [42]. At the same time, they used restraining information flow as a coping strategy to handle stressful situations [43]. In addition, this study shows that older adults are sensitive to messages perceived as stigmatizing and to what they perceive as a lack of logic in recommendations, showing the complexity of information needs in this population. Previous research indicates that older adults have a limited ability to detect fake news [44]; however, as they prefer traditional media, the government, and general practitioners as information sources, they are somewhat protected against online misinformation [45]. Therefore, how health information is presented to vulnerable groups is of great importance in preventing contamination during a pandemic, and it could be argued that there is a need to make special efforts.

Overall, the HBM model explained our data well. However, part of the Cues to action contradicted the results in Perceived susceptibility. This included how strongly their children influenced their behavior toward change and how they felt like a “culprit” when going against the recommendations. These reactions could be interpreted as not being *too* afraid of the virus after all and stressing the role of the family when implementing NPI. The HBM model is scarcely used in qualitative research and even less in an older adult population. However, the importance of cues to action from family and friends aligns with findings in a qualitative study on falls. These findings found that cues to action played an important role in older adults’ engagement in fall prevention [46]. This implies that the HBM has a role in qualitative research and should be considered to explore the potential for interesting findings further.

The difference between the first and second rounds of interviews was minor but not insignificant. In the follow-up interviews, we could see an increase in acceptance. The situation was no longer novel; the interviewees were used to the situation, and the promise of an upcoming vaccine probably eased the situation.

### Strengths and limitations

The strength of this study is, firstly, the many interviews we conducted during a short period in the initial months of the pandemic. We captured feelings and experiences in the moment of a novel and potentially frightening situation, along with the follow-up interviews about seven months later. This gave essential first-hand testimony from the population most vulnerable to the virus. Secondly, as Noone and Warner [17] and Perra [10] pointed out, there need to be more theory-driven studies on adherence to NPI, and we chose the HBM as an analytical tool, which can, therefore, be seen as a strength in this study. One limitation is that we conducted the interviews via telephone. Not seeing the other’s face can make it challenging to talk in-depth, especially about well-being, and natural pauses become rare and forced. However, it has been suggested that telephone interviews might increase feelings of anonymity, making respondents more relaxed and open [47]. Finally, the recruitment was made via social media and a pensioner’s organization’s information mail. As of this, we reached people with access to computer services, meaning we most likely have excluded those without these prerequisites. Thus, our participants were relatively homogenous in terms of socioeconomic status and living conditions. We would be cautious about transferring the results to older adults with significantly different prerequisites for maintaining their well-being. However, we managed to include men and women from various regions in Sweden.

## Conclusions

The overall conclusion from our study group is that these older persons, despite some potential barriers, are inclined to adhere to health-promoting recommendations during a crisis and have the necessary intention and resources required to adhere. The policy implications of our results, framed by the HBM, show that it is essential that consistency in communication, making an extra effort to explain in plain language, that information is easy to find, and availability to ask questions are aspects to consider when structuring health-promoting behavior in older adults. Further, much is gained if loneliness during isolation is mitigated since missing loved ones was seen as a potential barrier to adherence. Also, since we found that the grown-up children of older adults strongly influence their parents’ adherence, health-promoting interventions should possibly target relatives of older people to help bring the message.

Using HBM in qualitative research seems fruitful, especially regarding detection nuances in cues to action, and should be further explored. Knowledge gained from the COVID-19 pandemic should be internalized in developing recommendations for future pandemic outbreaks. Strengthening the preparedness and ability of those most at risk can yield significant benefits. Future research should focus on how older adults’ abilities, wants, and needs can be supported in crises.

### Electronic supplementary material

Below is the link to the electronic supplementary material.

Supplementary Material 1

Supplementary Material 2

## Data Availability

The datasets used and analysed during the current study are available from the corresponding author on reasonable request.
