# Supplementary material for: Understanding factors for adhering to health recommendations during COVID-19 among older adults - a qualitative interview study using health belief model as analytical framework
Source: BMC Geriatr. 2024 Jun 19;24:532. doi: 10.1186/s12877-024-05132-9 (PMC11188493; doi:10.1186/s12877-024-05132-9)
Supplement: Supplementary file 1 — Supplementary Material 1 [file 12877_2024_5132_MOESM1_ESM.docx]

***Appendix 1. Interview guide***

**Background question**

-How old are you?

**Introductory question**

-How has your everyday life been affected by the COVID-19 pandemic?

**Recommended restrictions regarding COVID-19**

-What rules/restrictions/instructions have you perceived or applied right now, concerning the coronavirus?

-How have you changed your habits to follow the recommendations?

-Have you continued to meet friends/children/grandchildren?

-How do you hang out today? How do you keep in touch?

-Have you continued to participate in arranged activities?

**About the COVID-19 information**

-How do you perceive the information about the new coronavirus?

-Has the information been available to you?

-Where did you get information from?

-What information did you get?

-What do you think about the information you received?

-Where would you like information from?

-What information have you been missing?

-How reliable do you think the information is?

-How do you assess the reliability of the information you access?

**The risk concerning COVID-19**

-How do you perceive the risk for yourself? Are you worried? Do you feel that you belong to the risk group?

-How do you perceive the risk to society? What consequences do you think the pandemic will have?

**About health concerning COVID-19**

-How do you experience your current health on a scale of 1-10?

-How do you experience your mental health during the current situation? How has it been affected and by what?

-Do you feel that something good has come out of this situation?

-Do you want to add something?
