# Supplementary material for: Understanding factors for adhering to health recommendations during COVID-19 among older adults - a qualitative interview study using health belief model as analytical framework
Source: BMC Geriatr. 2024 Jun 19;24:532. doi: 10.1186/s12877-024-05132-9 (PMC11188493; doi:10.1186/s12877-024-05132-9)
Supplement: Supplementary file 2 — Supplementary Material 2 [file 12877_2024_5132_MOESM2_ESM.docx]

***Appendix 2. Table of analyses.***

| **Example of the analytical process.** | **Meaning units** | **Categories** |
| --- | --- | --- |
| I am not especially afraid of dying, but I would like to live on | Not afraid, but don't want to die | Perceived susceptibility to and severity of the health threat |
| I can’t say that I a feel that the risk is enormous, but I am carful anyway. I really don’t want to catch it, especially not if I would get as sick as some do. But I don’t walk around worrying about getting sick al the time | Not a huge risk, but careful anyway |  |
| Well, I don’t know. It is dangerous and all, people die. But you die from colds as well. And, what’s it called, influenza. This one (COVID-19) is worse but many that die have underlying health problems that has an impact | Other things are worse |  |
| If we want an end to this, we need to follow the recommendations. We will just have to wait and see. Yes, I do, I feel really healthy myself. But I would not want to catch it (the virus) anyway. I think that would be awful and that's why we both stay at home. | Feel healthy but don't want to catch it |  |
| But I'm not worried about my health, I have to say. I'm not afraid of dying. It doesn't feel terrible, actually. On the other hand, it seems like a terrible, terrible way of dying, all alone and so...So it is not something you want of course | Not worried, but a terrible way of dying |  |
| My husband is ten years older than me. He is more in the risk zone, but at least we don’t walk around in panic. That’s not living | Worry for others |  |
| Well, I’m not…scared, but one has to be careful | Not scared, but careful |  |
